# Modern Image-Guided Surgery: A Narrative Review of Medical Image Processing and Visualization

**DOI:** 10.3390/s23249872

**Published:** 2023-12-16

**Authors:** Zhefan Lin, Chen Lei, Liangjing Yang

**Affiliations:** 1 School of Mechanical Engineering, Zhejiang University, Hangzhou 310030, China; zhefan.21@intl.zju.edu.cn; 2 ZJU-UIUC Institute, International Campus, Zhejiang University, Haining 314400, China; chen2.22@intl.zju.edu.cn

**Keywords:** image-guided surgery, medical image processing, 3D visual interface, augmented/mixed/virtual reality, surgical navigation

## Abstract

Medical image analysis forms the basis of image-guided surgery (IGS) and many of its fundamental tasks. Driven by the growing number of medical imaging modalities, the research community of medical imaging has developed methods and achieved functionality breakthroughs. However, with the overwhelming pool of information in the literature, it has become increasingly challenging for researchers to extract context-relevant information for specific applications, especially when many widely used methods exist in a variety of versions optimized for their respective application domains. By being further equipped with sophisticated three-dimensional (3D) medical image visualization and digital reality technology, medical experts could enhance their performance capabilities in IGS by multiple folds. The goal of this narrative review is to organize the key components of IGS in the aspects of medical image processing and visualization with a new perspective and insights. The literature search was conducted using mainstream academic search engines with a combination of keywords relevant to the field up until mid-2022. This survey systemically summarizes the basic, mainstream, and state-of-the-art medical image processing methods as well as how visualization technology like augmented/mixed/virtual reality (AR/MR/VR) are enhancing performance in IGS. Further, we hope that this survey will shed some light on the future of IGS in the face of challenges and opportunities for the research directions of medical image processing and visualization.

## 1. Introduction

Image-guided surgery (IGS) is a form of computer-assisted navigation surgery that focuses on processing image data and converting it into information for surgical systems or for visual displaying in the surgeon’s view. This process involves the timely tracking of targeted sites on the patient and the visualization of surgical tool motion, usually guided by a combination of a preoperative surgical plan and model with intraoperative imaging and sensing [1]. This innovative technology has a huge market potential, as it is expected to reach a value of USD 5.5 billion by 2028, growing at a compound annual growth rate of 5.4% during the forecast period [2]. The subject of IGS technology has substantial clinical relevance and is especially paramount to the development of minimally invasive procedures. While the continuous quest of improving surgical outcomes has led to rapid progress in sophisticated surgical techniques, characterized by less and smaller incisions to minimize the invasiveness of the procedures, its limited and non-direct visual access are inevitably making the procedures harder to perform. IGS technology aims at addressing these practical challenges by providing the surgeon with visual information augmentation from pre- and intraoperative imaging. By using fluorescence imaging during surgery, surgeons can see tumors and/or healthy tissues around them in real time, which helps them to remove tumors more accurately and avoid any mistake detrimental to the patients [3].

Medical image processing is an essential step that obtains and manipulates digital images of the patient body for medical diagnostic and treatment purposes. Medical image processing includes the processes of enhancing, segmenting, and registering the medical images using various algorithms and techniques. Image enhancement improves the quality and contrast of the images by removing noise, artifacts, or distortions. Image segmentation partitions the images into regions of interest, such as the organs, tissues, or lesions. Image registration aligns and fuses multiple images from different modalities or time points. The image processing pipeline is extremely crucial for IGS, which includes the use of preoperative and intraoperative images to guide surgical instruments and improve surgical outcomes. However, for the processed medical image data to be useful in IGS and translated to treatment benefits, the data need to be well visualized by surgeons. Image visualization displays intuitive images in two-dimensional (2D) or three-dimensional (3D) formats that can be interacted with by the user.

Medical image visualization is the process of presenting complex and multidimensional data in a clear and understandable way that can support clinical decision making and research. One of the challenges of medical image visualization is creating realistic and interactive representations of the data that can enhance the user’s experience and understanding. Immersive technology, such as augmented/mixed/virtual reality (AR/MR/VR), is a promising solution that can provide a sense of presence and immersion in a virtual or augmented environment. It differs in the views of the user. AR gives a view of the real world with an overlay of digital elements. VR gives a view of a fully immersive digital environment and the user can interact with it [4]. MR is a combination of AR and VR such that the user, real world, and virtual reality can interact with each other in real time [5]. Immersive technology has been integrated with surgical workflows for various purposes, such as preoperative planning, intraoperative navigation, and surgical training. However, there are also limitations and challenges that need to be addressed, such as user interaction, data quality, ethical issues, and technical feasibility.

Despite representative reviews and surveys in IGS being widely available, the focuses are typically specific to the surgical procedures or the organs being operated on. Our review focuses on analyzing navigation systems in general across a representative range of applications. For example, Ryan et al. evaluated IGN’s value in improving surgical accuracy and clinical outcomes specifically in spinal surgery [6]. Donovan et al. summarized current and developing techniques in surgical navigation for head and neck surgery [7]. DeLong et al. assessed the status of navigation in craniofacial surgery [8]. Du et al. performed a meta-analysis on the variation in pedicle screw insertion among 3D FluoroNav, 2D FluoroNav, and computed tomography-based navigation systems [9]. Although a few reviews have also discussed the navigation system in general as a whole, there is an inadequate number of reviews addressing and discussing the process methodology. Uli Mezger et al. reviewed a short history and the evolution of surgical navigation, as well as technical aspects and clinical benefits [10]. Arthur Randolph et al. reviewed equipment and operative setups [1]. Unlike these reviews, our review focuses on analyzing the methods used in image processing. It is important to note that the effectiveness and applicability of these methods may vary depending on specific use cases. For this reason, it is not in the scope of interest of this narrative review to compare the performance of existing methods.

Visualization techniques are drawing much attention for their intuitive interpretation of and including interaction with visual information. The focus of our review is not to summarize the technology or details of specific techniques; rather, we focused on the benefits of intuitive 3D information and the new visualization interfaces. Interested readers can refer to Preim’s review [11] for perceptually motivated 3D medical image data visualization and to Zhou’s review [12] for different types of visualization techniques based on data types, modalities, and applications.

Clinicians are typically interested in the kind of equipment and the methods that contribute to the setting up and realization of an effective navigation system. Available resources in the literature typically focus on the technical details of the imaging mechanism and principles which are often not well streamlined towards information retrieval for readers in the medical domain. In addition, there are promising technologies that have not yet been well introduced to medical communities like the AR/MR/VR technologies, which enrich the effects of existing image modalities and methods of viewing in IGS. This narrative review aims to fill the gap between the current system and MIA methods including a timely discussion of frontier technologies like the AR/MR/VR interface in the application of IGS.

**Figure 1:**
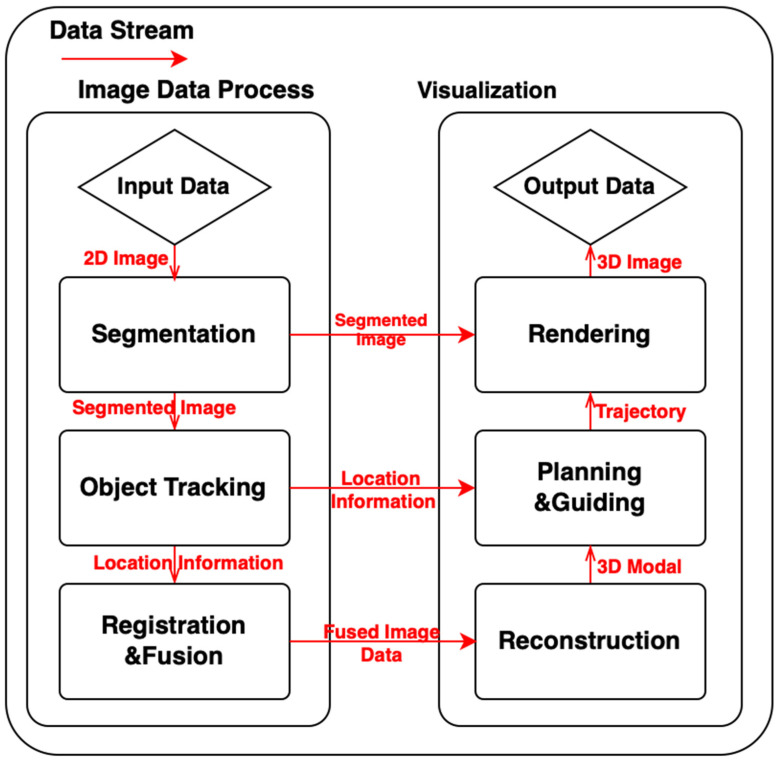
Image Data Stream in Surgical Navigation Workflows.

**Figure 2:**
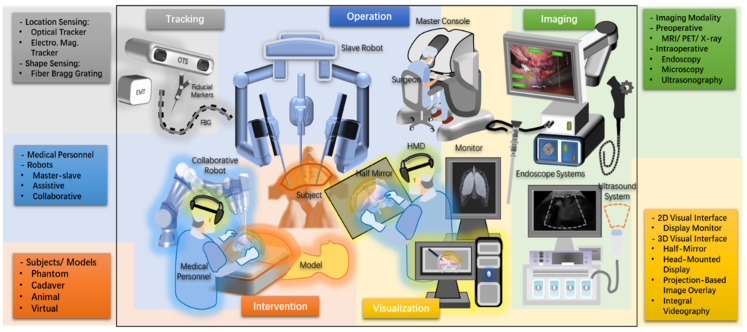
Diagram of the Surgical Navigation System.

The whole pipeline for the navigation system follows the processing of medical image data streams. Figure 1 shows the workflows that the image data stream goes through and how these workflows relate to each other in a navigation system. We also divided the surgical navigation system into five parts: tracking, visualization, intervention (subjects and the environment), operation (medical team and robotic systems), and imaging (medical image modalities), as shown in Figure 2. It provides an abstract image of a surgical navigation system, and we will use cases to show what methods they used and how they set up a surgical navigation system in the following sections. Methods of image data processes used in navigation systems will be introduced in Section 3.1. Methods and interfaces of visualization will be introduced in Section 3.2.

## 2. Materials and Methods

This narrative review was performed on publications between 2013 and 2023 using the databases Web of Science™, Scopus, and the IEEE Xplore^®^ Digital Library. The search string and number of papers are listed in Table 1. For IEEE Xplore^®^, the filters of “2013–2023”, “Conferences” and “Journals” were used. These papers were further filtered by exclusion criteria: (1) no English version; (2) duplicated; (3) irrelevant; and (4) unavailable. We also applied a snowballing search methodology using the references cited in the articles identified in the literature search. There were 39 representative papers with a complete navigation system between 2019 and 2022, as summarized in Section 3.2. Additional papers on classical image processing techniques like segmentation and 3D reconstruction before 2013 were also included.

## 3. Results

### 3.1. Medical Image Processing

Table 2 shows the summary of methods used in the segmentation, tracking, and registration part of surgical navigation systems. For segmentation, traditional methods and learning-based methods are both widely used while some auto-segmentation frameworks are becoming popular. For tracking, an electromagnetic tracker (EMT) and optical tracking system (OTS) are mostly used while some researchers have tried learning-based methods or SLAM-based methods. For registration, extrinsic methods like fiducial markers or landmarks and intrinsic methods like iterative closest point (ICP) or coherent point drift (CPD) are the main methods.

#### 3.1.1. Segmentation

Medical image segmentation is a process of dividing medical images into regions or objects of interest, such as the organs, bones, tumors, etc. It has many applications in clinical quantification, therapy, and surgical planning. Various methods have been proposed for medical image segmentation, including traditional methods based on boundary extraction, thresholding, and region growing [44,45,46], which are still popular among researchers. However, medical image segmentation faces some unique challenges:

- A lack of annotated data: Medical images are often scarce and expensive to label by experts, which limits the availability of training data for supervised learning methods.
- Inhomogeneous intensity: Medical images may have different contrast, brightness, noise, and artifacts depending on the imaging modality, device, and settings, which make it hard to apply a single threshold or feature extraction method across different images.
- Vast memory usage: Medical images are often high-resolution and 3D, which requires a large amount of memory and computational resources to process and store.

To address the above listed challenges, some researchers have proposed a multi-agent system (MAS). By forming a collection of individual agents that use the appropriate methods for different targets, MAS can handle complex segmentation problems. For example, Chitsaz et al. proposed a MAS composed of a moderator agent and several local agents that handle thresholding methods to segment CT images [47]. Bennai et al. proposed two organizations of agents to carry out region growing and refinement to segment tumor-in-brain MR images [48]. Moreover, due to the locality and stochasticity of local agents and the cooperation among them, the MAS approach is generally more robust than single-method approaches. The potential of handling a large number of images allows for fast segmentation. However, MAS usually requires prior knowledge and parameter estimation to initialize the agents. Some improved approaches can avoid prior knowledge [49,50,51,52] or parameters estimation [53,54]. Moreover, some research groups have also combined the MAS idea with reinforcement learning (RL). Liao et al. modeled the dynamic process of iterative interactive image segmentation as a Markov decision process and solved it with a multi-agent RL, achieving state-of-the-art results with the advantages of less interactions and a faster convergence [55]. Allinoui et al. proposed a mask extraction method based on multi-agent deep reinforcement learning and showed convincing results on CT images [56].

In recent years, thanks to the fast development of machine learning, most researchers have focused on learning-based methods that use deep neural networks to automatically learn features and segmentations from data [57,58,59,60,61,62]. Among these methods, U-Net is one of the most popular and widely used architectures for medical image segmentation due to its flexibility, optimized modular design, and success in all medical image modalities [57]. Several extensions and variants of U-Net have been developed to improve its performance and adaptability for different tasks and modalities [63,64]. Other networks, such as graph convolutional networks (GCNs) [58], variational autoencoders (VAEs) [59], recurrent neural networks (RNNs) [60,61], class activation maps (CAMs) [62], and so on are also used due to their advantages and applicability. In general, learning-based methods have certain advantages in segmentation accuracy and speed but also face limitations due to the scarcity of existing medical image datasets [65].

Moreover, some open-source frameworks have been proposed to facilitate the implementation and application of medical image segmentation methods for researchers and clinicians who lack experience in this field. For example, NiftyNet [66] is a TensorFlow-based framework that can perform segmentation on CT images; MIScnn [67] is a Python--based framework that supports state-of-the-art deep learning models for medical image segmentation; and 3DSlicer [34,35,68] is a software platform that can deal with 3D data or render 2D data into 3D.

#### 3.1.2. Object Tracking

Object tracking is essential for IGS that involves spatial localization of preoperative and intraoperative image data temporally. It can help locate the relative positions of surgeons, surgical tools, patients, and objects of interest, such as the diseased area and the surgical instruments, during IGS. External tracker tools, such as OTS for ex vivo tracking and electromagnetic tracking systems for in vivo tracking, are commonly used for this purpose. Optical tracking tools use an illuminator and passive marker spheres with unique retro-reflective surfaces that can be attached to any target and detected by the illuminator. Typically, the OTS will assign a frame of reference to facilitate calibration between devices’ coordinates and perform image-to-target registration [27,29,31,69]. By generating a defined EM field in which EM micro-sensors are tracked, rigid, and flexible, medical instruments embedded within these sensors can be tracked without obstruction. Researchers have usually used EMT for deep in vivo organ tracking and movement of the transducer [40,70,71,72,73]. By using external tracker tools, object tracking can achieve millimeter or sub-millimeter accuracy, but additional tools could mean limitations in medical use or incur extra expenses.

**Figure 3:**
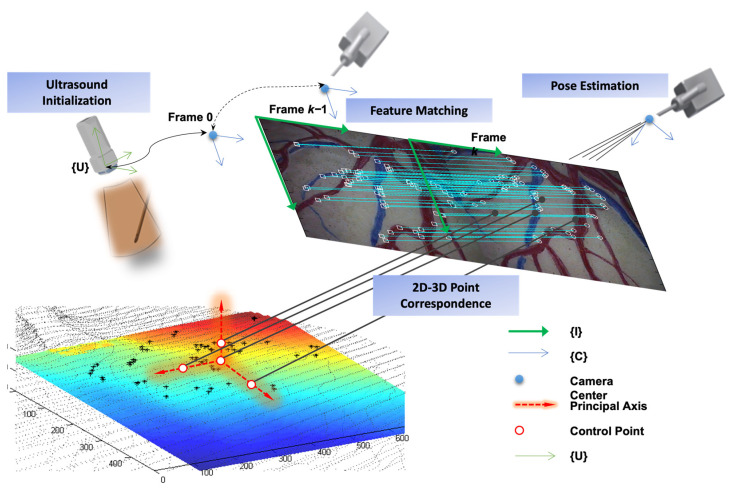
Combination of ultrasound localization and endoscopic vision-based pose estimation resulting in timely tracking that is cumulatively error-free.

Image-based object tracking is another approach that is widely used. It involves detecting and tracking objects in a sequence of images over time. Many features, strategies and state-of-the-art camera-based visual tracking methods have been reviewed and surveyed in [74,75,76,77,78,79]. In the medical domain, vision-based and marker-less surgical tool detection and tracking methods were reviewed in [80,81,82]. Other object tracking methods based on intraoperative imaging modalities include fluoroscopy-based [83,84], ultrasonography-based [85,86], and hybrid multimodalities [87,88], which combine ultrasound and endoscopic vision-based tracking as shown in Figure 3. In [88], the more accurate ultrasound-based localization is used for less frequent initialization and reinitialization while endoscopic camera-based tracking is used for more timely motion tracking. This hybrid form of motion tracking overcomes the inevitable cumulative error associated with vision-based pose estimation of a moving endoscope camera by triggering 3D ultrasound reinitialization, which can be conducted at less frequent intervals due to slower but cumulative error-free localization.

However, image-based object tracking still faces challenges such as low image quality, object motion, and occlusion. High distortion or artifacts of medical images pose greater challenges in object recognition, especially, for medical purposes where requirements for accuracy, reliability, and effectiveness are highly demanding. Some examples of these methods have used deep learning to detect and segment surgical tools in endoscopic images [89], convolutional neural networks (ConvNets) to track surgical tools in laparoscopic videos [90], or a combination of a particle filter and embedded deformation to track surgical tools in stereo endoscopic images [91]. Although there is room for improvement in terms of accuracy and real-time performance, the application of state-of-the-art image-based object tracking methods in surgical navigation is still an open research problem.

#### 3.1.3. Registration and Fusion

Registration is a key process in medical image analysis that involves aligning different coordinate systems that may arise from different perspectives, modalities, or techniques of data acquisition. Depending on whether the alignment can be achieved by a simple transformation matrix or not, registration can be classified into rigid or non-rigid types. Whereas rigid transformation can only handle rotation, scaling, and translation, non-rigid transformation allows for local warping and deformation of the images to achieve alignment. The image registration procedure involves finding relevant features in both volumes, measuring their alignment with a similarity metric, and searching for the optimal transformation to bring them into spatial alignment. And that is where deep learning comes in. Refs. [92,93] surveyed the recent advances and challenges of deep learning methods for medical image registration. Despite the lack of large datasets and a robust similarity metric for multimodal applications, researchers have recently used deep learning as a powerful and convenient tool for fast and automatic registration. For example, Balakrishnan et al. proposed VoxelMorph, a fast and accurate deep learning method for deformable image registration that learns a function to map image pairs to deformation fields, and can be trained in an unsupervised or semi-supervised way on large datasets and rich deformation models [94]. Vos et al. introduced the Deep Learning Image Registration (DLIR) framework, a novel method for unsupervised training of ConvNets for affine and deformable image registration using image similarity as the objective function. The DLIR framework can perform coarse-to-fine registration of unseen image pairs with high speed and accuracy, as demonstrated on cardiac cine MRI and chest CT data [95].

The main goal of registration is to find correspondences between features that represent the same anatomical or functional structures in different coordinate systems. Fusion is a related process that involves displaying data from different coordinate systems in a common one for visualization or analysis purposes. Often, registration and fusion are performed simultaneously to facilitate the integration of multimodal data. For example, Chen et al. used a unified variational model to fuse a high-resolution panchromatic image and a low-resolution multispectral image into the same geographical location [96].

**Figure 4:**
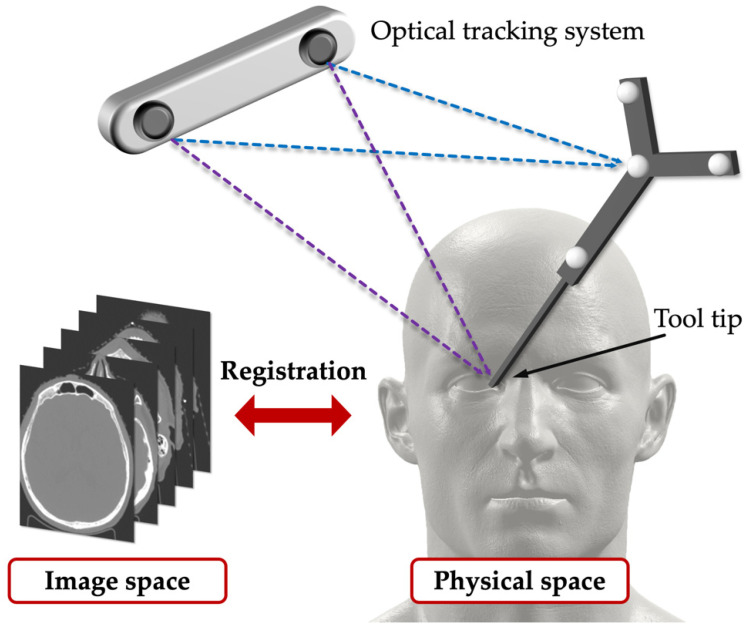
Overview of the calibration and registration using OTS.

In a surgical navigation system, surgeons require high accuracy, high confidence, and fast and robust registration methods. And one of the challenges of registration in surgery is to deal with non-rigid deformations that may occur during surgery or due to patient movement. To overcome this difficulty, researchers often use landmarks as salient features that can be easily identified and matched in different images. For instance, Jasper et al. proposed a navigation system that used landmark registration between a preoperative 3D model and an intraoperative ultrasound image to achieve active liver compensation with an accuracy below 10 mm [70]. Another example of using landmarks is the OTS, described in Section 3.1.2, which can also provide the landmark function. As shown in Figure 4, rigid point-based registration is performed between the physical space and the image space by using the OTS to track the surgical tool and measure the points in the physical space, and by using software to segment the points in the image space. For example, Sugino et al. used the NDI system and 3D Slicer to set up a surgical navigation system that collected data [18]. However, landmarks are hard to place in some conditions, such as the brain or the lung. Non-rigid registration between intraoperative images is required and also a challenging problem in medical image analysis, especially for organs that undergo large deformations during surgery.

#### 3.1.4. Planning

One of the essential tasks for surgeons, especially in craniofacial procedures, is to obtain high-quality information on the patient’s preoperative anatomy that can help them strategize and plan the surgical procedure accurately [97]. For instance, in tumor surgery, it is crucial to view the structure and morphology of the hepatic vessels and their relation to the tumors [98]. This 3D information can be derived from direct clinical measurements taken from physical models [99] or from digital models reconstructed from volumetric images such as CT or MR. In order to achieve this, segmentation, tracking, and registration techniques are employed to enable surgeons to see the surgical tools overlaid on the patient’s anatomy and even to see through obstructions and locate the targets. Based on this information, surgeons can plan the optimal route for surgery preoperatively and guide the surgical tool intraoperatively with the assistance of computer software. For example, Han et al. presented a method to automatically plan and guide screw placement in pelvic surgery using shape models and augmented fluoroscopy [100]. Li et al. presented a method to automatically plan screw placement in shoulder joint replacement using cone space and bone density criteria [101]. Surgical planning can improve the accuracy, safety, efficiency, and quality of surgery, especially for complex or minimally invasive cases. However, it can be time-consuming, costly, and technically challenging to produce accurate and reliable surgical plans because it usually requires a physical model to simulate the surgery. For example, Sternheim et al. used a Sawbones tumor model to simulate the resection of a primary bone sarcoma and reduced the risk of a positive margin resection [102].

In addition to traditionally used physical models in surgical planning, 3D imaging and virtual surgical planning (VSP) have become increasingly popular in orthognathic surgery in many regions of the world [103]. VSP requires 3D models that are usually reconstructed from volumetric images and need rendering procedures to be displayed on a screen. Advances in these computer-aided technologies have opened up new possibilities for VSP in craniofacial surgery [104]. VSP can provide more anatomically based and surgically accurate simulation of the procedure, enable a more interactive and collaborative planning process, and improve the predictability, precision, and outcomes of surgery. A usability study of a visual display by Regodić et al. reported that clinically experienced users reached the targets with shorter trajectories using VSP [105]. And Mazzola et al. showed that VSP reduced the time and maintained the cost and quality of facial skeleton reconstruction with microvascular free flaps [106].

### 3.2. Visualization

#### 3.2.1. 3D Reconstruction and Rendering

3D reconstruction in this paper means a process of generating 3D models from image slices/sequences. Rendering is an interactive process that allows the observer to adjust the display parameters to depict the point of interest most intuitively.

Accurate and clear 3D models for the visualization of anatomical structures of their patients is important for radiologists and surgeons. Supported by computer vision, tomographic reconstruction techniques for CT and MRI have been well developed in the past few decades and can provide high-quality visualization of human anatomy to help medical diagnostics. Nowadays, many platforms and types of software provide automatic reconstruction and rendering procedures, for example, ParaView, Seg3D, SynGO, Mimics, 3D Slicer, and so on. Usually, functionalities like auto-segmentation are also provided. Radiological imaging like CT and cone beam computed tomography can provide high-resolution and high-contrast images. However, due to their ionizing nature, they also pose the risk of radiation exposure to patients. Using low-dose ones on the other hand causes image quality degradation. Magnetic resonance imaging can provide non-invasive and non-radiation images, but they are also affected by some factors, such as metal objects, gas, bones, tissue depth, and background noise.

To address these issues, many researchers use deep neural networks to generate high-quality or complementary data [107,108,109]. The impressive performance of CNN-based low-dose CT restoration [110,111] has stimulated more research on deep learning methods for image reconstruction. Ref. [112] proposed a new algorithm that uses discriminative sparse transform constraints to reconstruct low-dose CT images with better quality and less noise by combining the advantages of image-compressed sensing reconstruction and a differential feature representation model and avoiding the drawbacks of the classical methods that depend on a prior image and cause registration and matching problems. Ref. [113] proposed a new deep learning network that uses a noise estimation network and a transfer learning scheme to adapt to different imaging scenarios and denoise low-dose CT images with better quality. Deep learning-based MR image reconstruction methods are plentiful, such as FA-GAN [114], FL-MRCM [115], U-Net [116], and so on. In the meantime, 2D X-rays, which are cost-effective, widely available, and expose patients to less radiation, can also be used to reconstruct 3D images with some methods proposed by researchers [117]. In short, generating and viewing 3D models for diagnostic purpose is a common phenomenon.

**Figure 5:**
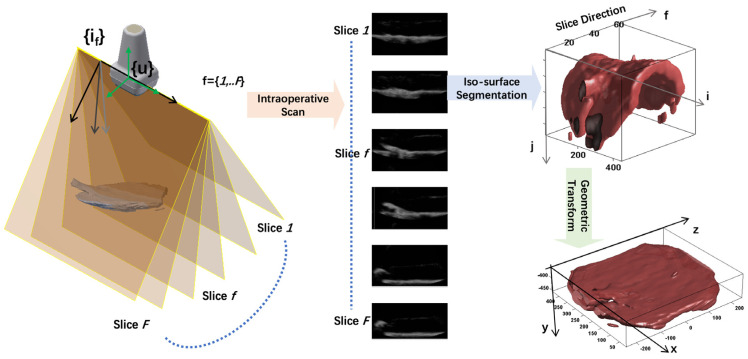
Using intraoperative two-dimensional (2D) ultrasound imaging with known spatial information to reconstruct a three-dimensional (3D) model [134].

Apart from 3D model reconstruction from diagnostic imaging, there are also dynamic 3D image reconstruction applications based on intraoperative imaging modalities. Ultrasound scanning, being a common intraoperative imaging modality, can be used to carry out 3D reconstruction [118,119]. Other than using a 3D ultrasound transducer that could acquire a 3D surface directly, intraoperative 2D ultrasound imaging can also reconstruct 3D models with known spatial information of the scan slice as illustrated in Figure 5 Reconstruction can subsequently be conducted after segmentation of the 3D surface based on the intensity of the ultrasonography, as illustrated in the same figure showcasing the 3D reconstruction of a placenta in a fluid medium [120]. Recent work in relation to 3D ultrasound reconstruction has also explored promising machine learning-based approaches [121,122]. Endoscopic camera-based image reconstruction is another commonly used approach for intraoperative reconstruction of 3D structures in the scene [123,124]. Most of the papers found applied techniques in photogrammetry for intraoperative mapping and surface reconstruction [125,126,127,128]. While these methods are mainly passive, i.e., relying purely on visual landmarks or interest points in the scene, there are also active camera-based approaches that cast structured lighting to the scene for surface reconstruction [129,130]. Camera-based approaches are promising for several clinical applications, including 3D reconstruction of the lumen in capsule endoscopy [131,132]. Some other methods involve a hybrid combination of ultrasonography and endoscopy for 3D reconstruction [87,88,133].

Usually, surgeons and researchers visualize images or volumes on a screen and operate using keyboards. However, with the help of AR/MR/VR technology, researchers can also augment real surgical scenes with the rendering of 3D models. Reconstruction and rendering are integral to IGS, consisting of sophisticated modern user interfaces and visual media.

#### 3.2.2. User Interface and Medium of Visualization

**Figure 6:**
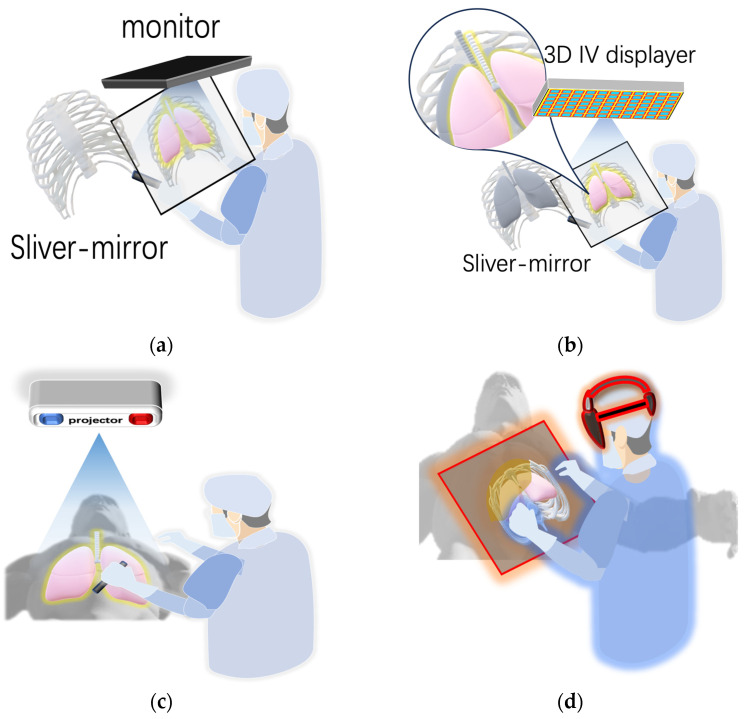
Diagram of (**a**) half-mirror, (**b**) integral videography, (**c**) image overlay, and (**d**) head-mounted display.

While reconstruction and rendering provide plentiful 3D spatial image data, visualizing image-based navigational information through a 2D screen imposes problems in hand–eye coordination and depth perception [135]. Merging real scenes and virtual images is one of the solutions. Several display technologies, including half-mirror, projection-based image overlay, and integral videography (IV), can show fused real and virtual data in real time, as shown in Figure 6. Half-mirror is a technique that uses a half-silvered mirror or a transparent monitor to reflect a virtual image onto the viewer’s eyes, while allowing them to see through the mirror or monitor to observe the real environment. With the advantages of being realistic, immersive, versatile, and energy-efficient, half-mirror is widely used in surgical navigation [136,137,138]. IV is a technique that uses, captures, and reproduces a light field using a 2D array of micro-lenses to create autostereoscopic images. With the advantages of being autostereoscopic, natural, and parallax-rich, IV is widely used in displaying 3D anatomical structures or surgical plans inside the patient [139,140,141]. Projection-based image overlay is a technique that uses a projector to display a virtual image onto a screen or a real object, such as a wall or a table. With the advantages of being simple, scalable, and adaptable, projection-based image overlay is widely used in projecting guidance information or registration markers on the patient [142,143].

**Figure 7:**
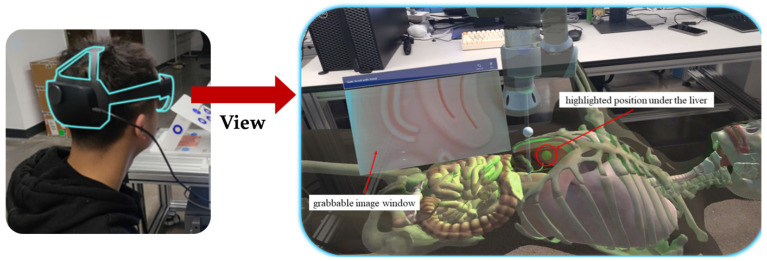
Example of seeing a 3D model in HoloLens [144].

**Figure 8:**
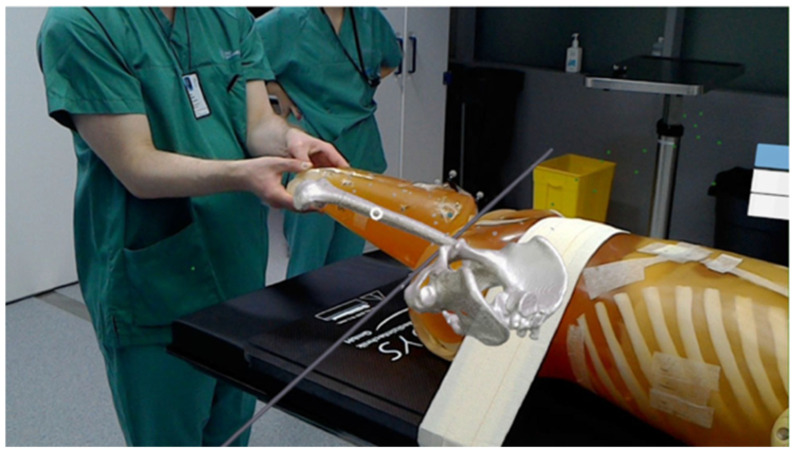
Patient phantom seen in HoloLens while the surgeon manipulates the limb [27].

In contrast to these techniques, which rely on external devices to create the AR effect, another approach is to use wearable devices that can directly display the virtual images in the user’s view. Head-mounted displays (HMDs) like HoloLens are innovative devices that can augment many kinds of surgery. They can not only display images and 3D models on the user’s view, as shown in Figure 7 [144], but also use the HMD’s image as an image source for various purposes. All tasks of surgical navigation, such as segmentation, object tracking, registration, fusion, and planning, can be performed on HMDs. For example, Teatini et al. used HoloLens to provide surgeons with the illusion of possessing “X-ray” vision to visualize bones in orthopedics [27], as shown in Figure 8. Teatini used Polaris Spectra and NDI as a means of optical tracking and optical markers to conduct rigid registration. By conducting an evaluation study on two phantoms, Teatini demonstrated that the MR navigation tool has the potential to improve diagnostic accuracy and provide better training conditions. Furthermore, HMDs like HoloLens have other functions that can be utilized in the surgical condition including gesture recognition and audio control. These functions can enhance the convenience for and efficiency of the surgeon. Nishihori et al. accessed a contactless operating interface for 3D image-guided navigation and showed some benefits [145]. However, Nishihori needed additional devices like Kinect to perform gesture recognition and use voice recognition software. HMDs like HoloLens are an integrated interface that incorporate these functions and similar systems can be developed based on them. Therefore, HMDs are a promising technology that can revolutionize surgical practices and outcomes.

#### 3.2.3. Media of Visualization: VR/AR/MR

**Figure 9:**
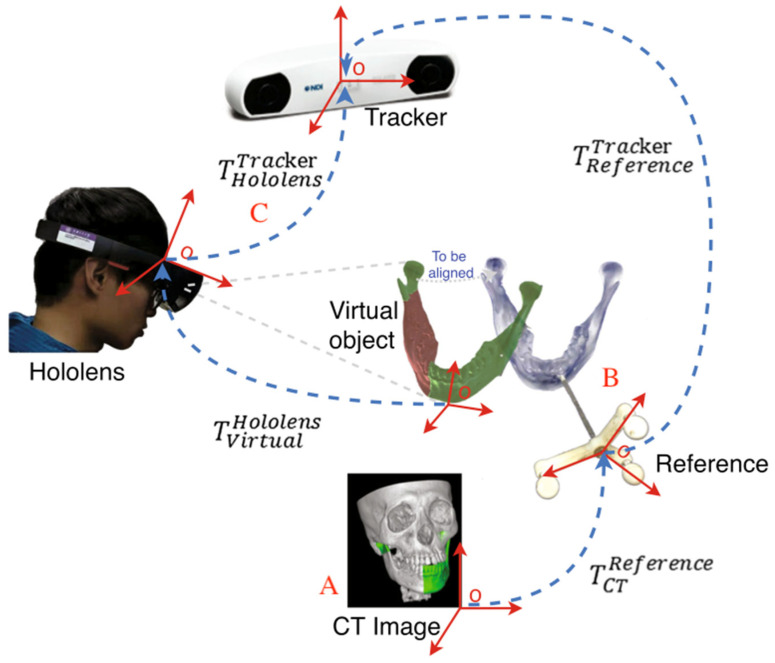
Example of augmented reality (AR)-based surgical navigation system used in [29].

VR/AR/MR technologies are emerging fields that have attracted a lot of interest and attention in modern medicine. Many research groups have applied such technology in various domains, such as treatment, education, rehabilitation, surgery, training, and so on [146]. VR/AR/MR technologies differ in their degree of immersion and interaction, but they all aim to enhance the user’s experience by creating realistic and engaging environments [147]. One of the earliest applications of AR was to solve a simple problem: how to see the surgical monitor while instruments are inside patients. Yamaguchi et al. built a retinal projection HMD in 2009 to overlay the image and verify its accuracy [148]. Since then, AR technology has advanced significantly and has been used for more complex and challenging tasks. For example, Burström et al. demonstrated the feasibility, accuracy, and radiation-free navigation of AR surgical navigation with instrument tracking in minimally invasive spinal surgery (MISS) [149]. In Figure 9, Sun et al. proposed a fast online calibration procedure for an optical see-through head-mounted display (OST-HMD) with the aid of an OTS [29]. In this system, the whole procedure consisted of three steps: (1) image segmentation and reconstruction, as shown in Figure 9 (A); (2) point-based registration or an ICP-based surface matching algorithm, as shown in Figure 9 (B); and (3) calibration of the OST-HMD, as shown in Figure 9 (C). These examples show how AR technology can improve the accuracy and efficiency of surgical procedures.

**Figure 10:**
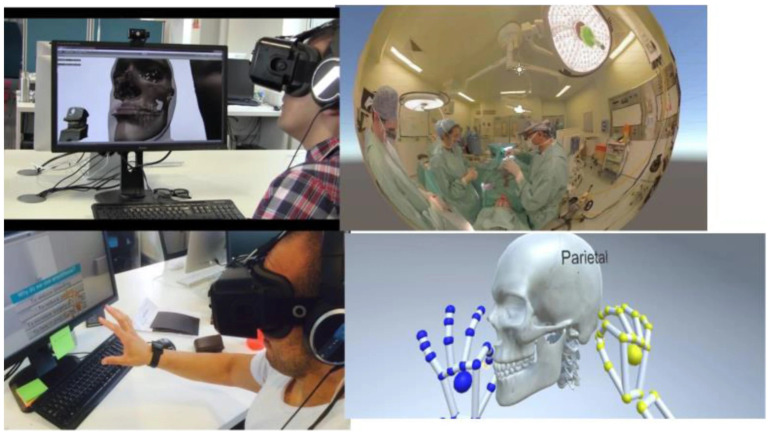
Example of a virtual reality (VR) simulator used in [153].

A VR simulator is a powerful tool that can be used for teaching or training purposes in medicine. Researchers have explored the use of VR simulators since the early 2000s, when they developed and evaluated various laparoscopic VR systems [150]. Khalifa et al. foresaw that VR has the ability to streamline and enhance the learning experience of residents by working synergistically with curriculum modalities [151]. Jaramaz and Eckman presented an example of a VR system using fluoroscopic navigation [152]. Nowadays, VR has been widely used in the education, training, and planning areas for different surgical specialties. For instance, in oral and maxillofacial surgery, VR has been utilized to improve the delivery of education and the quality of training by creating a virtual environment of the surgical procedure [153], as shown in Figure 10. Haluck et al. built a VR surgical trainer for navigation in laparoscopic surgery [154]. Barber et al. simulated sinus endoscopy using a VR simulator that combines 3D-printed models. They provided evidence that such a VR simulator is feasible and may prove useful as a low-cost and customizable educational tool [155]. These examples show how VR simulators can offer realistic and interactive scenarios for surgical education and training.

**Figure 11:**
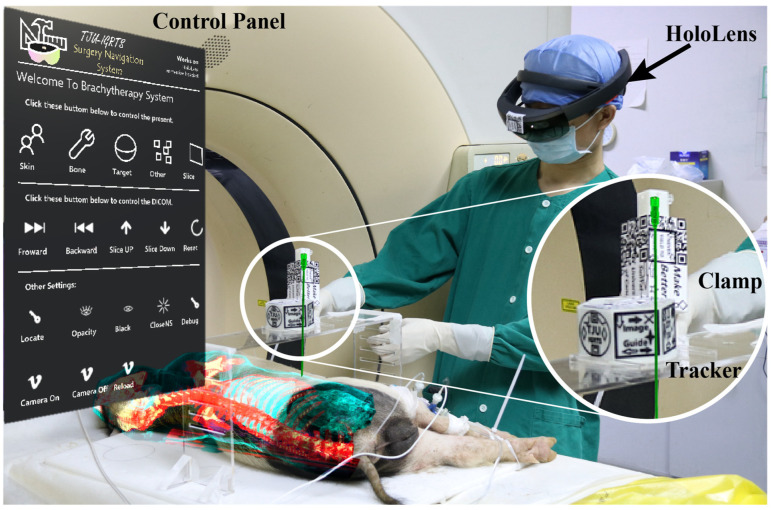
Example of a mixed reality (MR) navigation system used in [157].

MR technology has several features that make it ideal for image-guided navigation, such as a see-through view, spatial mapping, and an interactive interface. Several studies have evaluated and validated MR navigation systems for different surgical procedures and have shown positive results. For example, Martel et al. evaluated an MR navigation system in ventriculostomy and showed a 35% accuracy improvement [156]. Zhou et al. validated an MR navigation system for seed brachytherapy and showed clinically acceptable accuracy [157], as shown in Figure 11. Mehralivand et al. tested the feasibility of a VR-assisted surgical navigation system for radical prostatectomy and showed great usability [158]. Frangi et al. validated an MR navigation system for laparoscopic surgery and showed significant time saving [159]. Incekara et al. provided proof of concept for the clinical feasibility of the HoloLens for the surgical planning of brain tumors and provided quantitative outcome measures [160]. McJunkin et al. showed the significant promise of MR in improving surgical navigation by helping surgical trainees to develop mental 3D anatomical maps more effectively [161]. MR surgical navigation (MRSN) can aid doctors in performing surgery based on a visualized plan and achieving clinically acceptable accuracy [162]. MRSN is feasible, safe, and accurate for lumbar fracture surgery, providing satisfactory assistance for spine surgeons [163]. Therefore, MR technology is a promising tool that can enhance surgical performance and outcomes.

## 4. Discussion

In this narrative review, we have outlined the tasks of IGS and how they are related to the image data stream. We have followed the image data stream to illustrate how image data are used in each workflow: segmentation, tracking, registration, and planning. Image processing in each workflow is in-series but research groups in each workflow are independent from each other. Moreover, there are many research groups who are focused on setting up IGS systems using various methods and technologies. Nowadays, advanced methods are still being proposed in these areas and, in particular, methods based on AI are taking a prominent place. However, these methods are only focused on their own area and are hard to be applied to the IGS system. Finding out how to fill the gap between these methods and the system has led to a number of interesting research challenges related to satisfying the high accuracy, reliability, and effectiveness requirements of surgery. VR/AR/MR as a new type of visualization method show benefits and are an extension to IGS. They can provide realistic and interactive scenarios for surgical education, training, and planning. Some applications have been proposed but they are still in a primary state. As a solution, some software has been proposed to provide integrated functionality. For example, the 3D Slicer can automatically perform segmentation and reconstruction. But, state-of-the-art machine learning-based methods were not included because of customized models and databases. Therefore, there is a need for more research on how to integrate these methods into the software and the system.

Medical images have always been accompanied by security concerns because they carry private patient information. Currently, there exist various image security techniques like encryption, watermarking, steganography, etc. [164,165]. Since the image streams in the IGS system are only transmitted internally, security issues are not often involved.

Evaluating surgical navigation systems is an important and challenging task that requires appropriate performance metrics. The current performance metrics can be divided into three categories: outcome, efficiency, and errors [166]. Outcome metrics measure the quality and effectiveness of the surgical procedure, such as the accuracy of tumor removal or the preservation of healthy tissue. These metrics usually require the help of medical experts to mimic the metrics used to measure expertise. Efficiency metrics measure the speed and ease of the surgical procedure, such as the time consumed and the path length. These metrics can reflect the usability and convenience of the surgical navigation system. Error metrics measure the deviation and uncertainty of the surgical procedure, such as the percentage of error, deviation from target, or accuracy. These metrics can indicate the reliability and robustness of the surgical navigation system. For an IGS system, it is intrinsic to evaluate the registration and tracking part, which are essential for aligning and updating the image data with the surgical scene. According to [167], factors that contribute to the final evaluation of the whole system include the fiducial registration error (FRE), fiducial localization error (FLE), target registration error (TRE), overlay error (OR), and tool error (TE). These factors can quantify the accuracy and precision of the IGS system. Moreover, some qualitative evaluations have been proposed and usually given by surgeons to describe their subjective opinion on aspects such as comfort, confidence, satisfaction, and preference [168]. These evaluations can capture the user experience and feedback on the IGS system.

Choosing appropriate evaluation metrics is crucial for assessing the performance and validity of surgical navigation systems. The evaluation metrics should be aligned with the purpose and objectives of the research. For example, in [158], Mehralivand et al. wanted to evaluate the feasibility of interactive 3D visualization of prostate MRI data during in vivo robot-assisted radical prostatectomy (RARP). They chose outcome metrics such as blood loss, Gleason score, postoperative prostate-specific antigen (PSA), Sexual Health Inventory for Men (SHIM) score, and International Prostate Symptom Score (IPSS) as evaluation metrics to show the oncological and functional results of their system. In [159], Frangi et al. wanted to show the improvement in their MRSN system compared with LN-CT for laparoscopic surgery. They chose time consumption as an efficiency metric to show the speed and ease of their system. Outcome metrics and efficiency metrics usually have specific standards or need to be compared with other current approaches. In [156], Martel et al. showed a 35% improvement in accuracy for tip and line distances (13.3 mm and 10.4 mm to 9.3 mm and 7.7 mm) compared with conventional methods. In [148], Yamaguchi et al. showed 3.25% and 2.83% maximum projection error in overlaying virtual images onto real surgical stents using their retinal projection HMD system. Error metrics are widely used and usually required in the tracking of device tip and registration between preoperative and intraoperative images. Using multiple evaluation metrics is also common and can provide a more comprehensive assessment of the system. For example, Burström et al. used the accuracy of the device tip, angular deviation, time consumption, and user feedback to show the feasibility of an ARSN system in MISS [149]. It is plausible that maximum errors of 1.5–2 mm are acceptable in surgical navigation system by researchers. However, shifting from phantom or cadaver experiments to animal or human experiments can lead to an accuracy drop due to various factors such as tissue deformation, organ motion, and blood flow. For example, Zhou et al. reached a 0.664 mm needle location average error and 4.74% angle error in a phantom experiment and 1.617 mm and 5.574%, respectively, in an animal experiment [157]. Some researchers have also designed some evaluation models [169] for a HoloLens-based MRSN system based on analytical hierarchy process theory and ergonomics evaluation methods.

Despite not being widely used, HMD devices are attractive platforms for deploying AR/VR/MR surgical navigation systems, as they can provide low-cost, integrated, and immersive solutions for surgical navigation. Moreover, HMD devices can also enable contactless interaction systems, such as gesture control and voice control, which can enhance convenience for and efficiency of surgeons. However, transferring existing methods from conventional monitors to HMD devices is not a trivial task, as it requires adapting to different hardware specifications, user interfaces, and user experiences. Therefore, research and development on how existing methods can be better transferred to HMD devices is an important direction. It is envisioned that immersive technology will transform the way surgeons visualize patient information and interact with the future technologies in the operating room during IGS.

## 5. Conclusions

IGS is an evolving discipline that aims to provide surgeons with accurate and comprehensive information during surgical procedures. Traditional intervention requires surgeons to collect various types of image information from different modalities, such as CT, MRI, US, and so on. However, these modalities have limitations in terms of resolution, contrast, invasiveness, and cost. Moreover, integrating and visualizing these image data in a meaningful and intuitive way is a challenging task. IGS systems can overcome these limitations by enriching the information presented to the surgeons using advanced image processing and visualization techniques. However, the update of these systems has been limited by issues such as immersive accuracy and non-intuitive user interface. Integrated systems in the form of HMD have the potential to bridge these technology gaps, providing the surgeon with intuitive visualization and control. It is clear from this review that IGS systems have not yet reached maturity as technology and engineering develop further. While progress has been made in segmentation, object tracking, registration, fusion, planning, and reconstruction, combining these independent progresses into one system needs to be addressed. Visualization technologies like VR/MR/AR interfaces provide the possibility of an integration system to address the concerns of cost and complexity. HMD devices can also enable contactless interaction systems, such as gesture control and voice control, which can enhance convenience for and efficiency of surgeons. However, it is not easy to apply existing methods using conventional monitors to HMD devices, because they need to adjust to different hardware specifications, user interfaces, and user experiences. It is anticipated that the achievement of these research directions will lead to the development of IGS systems that will better support more clinical applications.

## Figures and Tables

**Table 1:** List of Search Strings and Databases.

Database	Search String	Number
Web of Science™	(TS = (surgical navigation)) AND ((KP = (surgical navigation system)) OR (KP = (image guided surgery)) OR (KP = (computer assisted surgery)) OR (KP = (virtual reality)) OR (KP = (augmented reality)) OR (KP = (mixed reality))OR (KP = (3D))) and Preprint Citation Index (Exclude—Database)	597
Scopus	((TITLE-ABS-KEY(“surgical navigation”)) AND ((KEY(“surgical navigation system”)) OR (KEY(“image guided surgery”)) OR (KEY(“computer assisted surgery”)) OR (KEY(“virtual reality”)) OR (KEY(“augmented reality”)) OR (KEY(“mixed reality”)) OR (KEY(“3D”))) AND PUBYEAR > 2012 AND PUBYEAR < 2024 AND (LIMIT-TO (SUBJAREA,”MEDI”) OR LIMIT-TO (SUBJAREA,”COMP”) OR LIMIT-TO (SUBJAREA,”ENGI”)) AND (LIMIT-TO (DOCTYPE,”ar”) OR LIMIT-TO (DOCTYPE,”cp”)))	1594
IEEE Xplore^®^	((“surgical navigation”) AND ((“surgical navigation system”) OR (“image guided surgery”) OR (“computer assisted surgery”) OR (“virtual reality) OR (“augmented reality) OR (“mixed reality”) OR (“3D”)))	146

**Table 2:** Summary of Categorized Methods in Surgical Navigation Systems.

Paper	Segmentation	Tracking	Registration
[13]	No	EMT ^1^	Rigid landmark
[14]	3D Slicer ^6^	EMT	PDM ^2^
[15]	Threshold	EMT	ICP ^3^/B-Spline
[16,17,18,19,20]	No	OTS ^4^	Fiducial markers
[21]	3D Slicer	No	Fiducial markers
[22]	Yes	No	ICP
[23]	3D Slicer	OTS	Surface-matching
[24]	Learning-based	No	ICP
[25]	No	Learning-based	Learning-based
[26]	Learning-based	No	No
[27,28]	Yes	OTS	Fiducial markers
[29]	Threshold/Region growing	OTS	ICP
[30]	Region growing	Visual–inertial stereo slam	ICP
[31]	Threshold	Learning-based	Super4PCS [32]
[33]	Yes	OTS	ICP
[34]	3D Slicer	EMT	Fiducial markers
[35]	Mimics ^6^	OTS	Anatomical landmark
[36]	Manual	No	ICP
[37]	No	Depth estimation	Learning-based [38]
[39]	EndoSize ^6^	No	Rigid intensity-based
[40]	Yes	EMT	ICP
[41]	Threshold	OTS	ICP
[42]	3D Slicer/Learning-based	No	ICP and CPD ^5^
[43]	Yes	Visual SLAM	Visual SLAM

^1^ EMT: electromagnetic tracker. ^2^ PDM: Philips Disease Management. ^3^ ICP: iterative closest point. ^4^ OTS: optical tracking system. ^5^ CPD: coherent point drift. ^6^ Public software: 3D Slicer, Mimics, EndoSize.

## Data Availability

Not applicable.
